# The Effects of High Concentrations of Vitamin C on Cancer Cells

**DOI:** 10.3390/nu5093496

**Published:** 2013-09-09

**Authors:** Seyeon Park

**Affiliations:** Department of Applied Chemistry, Dongduk Women’s University, 23-1 Wolgok-dong, Sungbuk-ku, Seoul 136-714, Korea; E-Mail: sypark21@dongduk.ac.kr or seypark21@hotmail.com; Tel.: +82-2-940-4514; Fax: +82-2-940-4193

**Keywords:** high concentrations of vitamin C, pharmacogenomics, pharmacoproteomics

## Abstract

The effect of high doses of vitamin C for the treatment of cancer has been controversial. Our previous studies, and studies by others, have reported that vitamin C at concentrations of 0.25–1.0 mM induced a dose- and time-dependent inhibition of proliferation in acute myeloid leukemia (AML) cell lines and in leukemic cells from peripheral blood specimens obtained from patients with AML. Treatment of cells with high doses of vitamin C resulted in an immediate increase in intracellular total glutathione content and glutathione-S transferase activity that was accompanied by the uptake of cysteine. These results suggest a new role for high concentrations of vitamin C in modulation of intracellular sulfur containing compounds, such as glutathione and cysteine. This review, discussing biochemical pharmacologic studies, including pharmacogenomic and pharmacoproteomic studies, presents the different pharmacological effects of vitamin C currently under investigation.

## 1. Introduction

There is increasing evidence that vitamin C (ascorbate) is selectively toxic to some types of tumor cells, functioning as a pro-oxidant [1,2,3]. Vitamin C at concentrations of 10 nM–1 mM induced apoptosis in neuroblastoma and melanoma cells [4] and was shown to be an important modulator for the growth of mouse myeloma cells in an *in vitro* colony assay [5]. Studies have established that the growth of leukemic progenitor cells from patients with acute myeloid leukemia (AML) and myelodysplastic syndromes (MDS) can be significantly modulated by vitamin C [6,7]. Intravenous (i.v.) administration of sodium 5,6-benzylidene-l-ascorbate (SBA) to inoperable cancer patients induced a significant reduction in tumor volume without any adverse side effects [8]. Furthermore, recent clinical studies have reported that manipulation of vitamin C levels *in vivo* can result in clinical benefit for patients with AML and solid tumors [9].

Recent pharmacokinetic studies reported that 10 g of ascorbate given by i.v. produced plasma concentrations of nearly 6 mM, which were 25-fold higher than concentrations resulting from the same oral dose [10,11,12]. Depending on the dose, as much as a 70-fold difference in plasma concentration was found between oral and i.v. administration [13]. Complementary and alternative medicine practitioners worldwide currently use ascorbate i.v. in patients, in part because there are no apparent harmful effects [14,15,16]. Physiological concentration of vitamin C is under 0.1 mM, while plasma vitamin C concentrations that cause toxicity to cancer cells *in vitro* (1 mM–10 mM, depending on cell lines) can be achieved clinically by intravenous administration, which means a high dose of vitamin C.

In this review, *in vitro* and *in vivo* studies are summarized, showing that ascorbate killed cancer cells (Table 1). In addition, the mechanisms and physiologic relevance under investigation are also described. The results suggest that doses of vitamin C induce oxidative stress in cancer cells. Our previous results indicated that treatment of malignant lymphocytic cell lines with vitamin C (0.25–1 mM) for 24 h led to a marked dose-dependent decrease of cell proliferation [17]. The responsive cell lines were human myeloid leukemia cell line HL-60, retinoic acid (RA)-sensitive acute promyelocytic leukemia (APL) cell line NB4 and RA-resistant APL cell line NB4-R1. Different types of leukemia cells, such as K562 (chronic myelogenous leukemia cell line) [17] and KG1 (myeloblast cell line) [18], were also responsive to vitamin C. A similar result was obtained with cells containing over 90% of blasts from patients with AML. In these cell lines, induction of apoptosis by vitamin C demonstrated a dose-dependent effect. In addition, vitamin C weakly induced apoptosis in ovarian cell lines, including SK-OV-3, OVCAR-3 and 2774 [17]. For many of the cancer cell lines, ascorbate concentrations caused a 50% decrease in cell survival. The half maximal concentration (IC_50_) values were less than 5 mM, and all tested normal cells were insensitive to 20 mM ascorbate [13].

**Table 1:** Effects of vitamin C treatment on cell survival [13,17,18].

Cell Line	IC_50_ (mM)
HL-60	0.33 ± 0.18 *
NB4	0.76 ± 0.14 *
NB4-R1	0.45 ± 0.24 *
NB4-R2	0.75 ± 0.3 *
KG1	0.79 ± 0.22 *
K562	0.5 ± 0.11 *
U937	0.3 ± 0.16 *
Normal Bone Marrow	1 ± 0.3 *
Patient with AML	0.84 ± 0.16 *
OVCAR	>10 *
SK-OV3	>10 *
JLP119	<1
MCF7	2
MB231	7
Hs587T	20
KLN205	<1
RAG	<2
CT26	4
B16	7
LL/2	11
Hs587Bst	>20
CCD34SK	>20
Human normal lymphocyte	>20
Human normal monocyte	>20

The IC_50_ values are means ± standard deviations from triplicate experiments. HL-60, human myeloid leukemia; NB4, NB4-R1, NB4-R2, human acute promyelocytic leukemia (APL); KG1, human myeloblast; K562, human chronic myelogenous leukemia; U937, human histiocytic lymphoma; OVCAR, SK-OV3, ovarian cancer; JLP119, human lymphoma; MCF7, MB231, Hs587t, human breast cancer; KLN205, mouse lung cancer; RAG, mouse kidney cancer; CT26, mouse colon cancer; B16, mouse melanoma; LL/2, mouse lung cancer; Hs587Bst, human normal breast cells; CCD34SK, human normal fibroblast cells. * IC_50_ value was determined using H^3^ incorporation proliferation assay for 24 h.

## 2. Molecular Mechanisms Induced by Vitamin C

In our previous study, vitamin C at concentrations of 0.25–2.0 mM significantly induced apoptosis in AML cell lines. Vitamin C induced oxidation of glutathione (GSH) to its oxidized form (GSSG). As a result, H_2_O_2_ accumulated in a concentration-dependent manner, in parallel with the induction of apoptosis. The direct role of H_2_O_2_ in the induction of apoptosis in AML cells was demonstrated by the finding that catalase could completely abrogate vitamin C-induced apoptosis [17].

A 30-min incubation of NB4 cells with 0–10 mM vitamin C resulted in its uptake in a concentration-dependent manner [17,19]. In accordance with its proposed pro-oxidant activity, vitamin C treatment reduced the GSH/GSSG ratio, which correlated with increased intracellular H_2_O_2_ in the NB4 cell line. Levine *et al.* suggested that the effect was due only to extracellular and not intracellular ascorbate, and that ascorbate-mediated cell death was probably due to protein-dependent extracellular H_2_O_2_ generation, via ascorbate radical formation from ascorbate as the electron donor [13].

Although H_2_O_2_ induced by ascorbate was first generated extracellularly, it is possible that it could diffuse across the plasma membrane into the intracellular space. Although studies in yeast and bacteria have shown that diffusion of H_2_O_2_ across membranes is limited, some reports have suggested that selected aquaporin homologues from plants and mammals can channel H_2_O_2_ across these membranes [20,21,22,23,24,25]. The susceptibility of cancer cells to ascorbate treatment might therefore be related to the permeability of hydrogen peroxide.

In our studies, vitamin C dramatically increased intracellular GSH oxidation and reactive oxygen species (ROS) levels within 3 h in a concentration-dependent manner. However, this GSH oxidation and the ROS accumulation did not last for a long period of time. After 3 h, the increase in GSH has been observed and hypothesized to be a defense mechanism [18]. No additional ROS accumulation resulted from the change in GSH. However, based upon our studies, the dramatic changes of intracellular oxidation state within 3 h seem to be enough to induce intracellular signaling. The studies showed that treatment with 1 mM vitamin C for only 30–60 min, followed by removal when replacing media, could induce apoptosis in both HL-60 and NB4 cells. This observation is consistent with initiation of apoptosis, resulting from generation of H_2_O_2_ after treatment with ascorbate. However, treatment with As_2_O_3_ resulted in less ROS accumulation than with vitamin C, and it was not in a concentration-dependent manner. However, ROS accumulation increased up to 24 h, with a long-lasting effect. Treatment with vitamin C combined with As_2_O_3_ increased ROS accumulation, as well as sustained the effect for up to 24 h. These results are consistent with cellular data showing that apoptosis induced by As_2_O_3_ is evident even at three days [26,27].

## 3. Proteomics Studies

Proteomics provides important qualitative information on post-translational modifications to proteins and quantitative data on protein expression in response to a particular stimulus. This information is particularly important when it provides data on early cellular events, such as the stimulus and signaling cascades triggered independently of protein neosynthesis. In accordance with its proposed pro-oxidant activity, vitamin C-mediated reduction in the GSH/GSSG ratio correlated with an intracellular H_2_O_2_ increase in the NB4 cell line [17,19]. This type of change in regional oxidation state could cause changes in the cellular milieu that could result in changes in protein structure. This is especially true of the oxidation state of cysteine sulfur, which is important for determining the tertiary structure of proteins. The immediate effects of cell stimuli are associated with protein post-translational modifications, such as phosphorylation, glutathionylation and cysteine oxidation. To study these early modifications, NB4 human leukemia cells were treated with 0.5 mM vitamin C and then analyzed by two-dimensional analysis. Approximately 240 different spots that were focused in a pH range of 4–7 were detected per sample.

After exposing cells to vitamin C, we observed one new spot, three intensified spots and five attenuated spots [19]. Each of these spots were excised, digested with trypsin and analyzed by matrix-assisted laser desorption/ionization-time of flight (MALDI-TOF). Peptide mass fingerprint analysis and non-redundant sequence database matching allowed the unambiguous identification of all of the analyzed species [19]. An important protein identified from the proteomic analysis was a thiol/disulfide exchange catalyst, protein disulfide isomerase (PDI), which was a marker of the effect of vitamin C on NB4 cells [19]. PDI belongs to the superfamily of thiol/disulfide exchange catalysts, which act as protein-thiol-oxidoreductase enzymes, sharing sequence homology with thioredoxin [28]. PDI is composed of four domains, which have similarities with thioredoxin folds (*i.e.*, a-b-b′-a′) [29]. In our study [19], the intensity of the spot corresponding to the PDI b subunit was decreased in vitamin C-treated cells as compared with control cells. These results demonstrated that thiol/disulfide exchange proteins are regulated in NB4 cells after vitamin C exposure. This is consistent with our study showing that intracellular GSH/GSSG exchange occurs shortly after vitamin C exposure [17].

When we measured cysteine uptake in leukemia cell lines exposed to vitamin C by using ^35^S-labeled-L-Cys in the media, the time-dependent rate of cysteine uptake in the cell culture increased significantly. The rate of uptake, determined under conditions without vitamin C, was very low. The glutathione synthesis inhibitor, buthionine sulfoximine, potently inhibited this increase, suggesting that incorporation of cysteine that corresponded to the amount of increased glutathione was mediated by glutathione synthesis. Overall, our results indicated that vitamin C-induced glutathione synthesis was accompanied by intracellular cysteine uptake.

Glutathione-S transferases (GSTs) are enzymes that catalyze the conjugation of electrophilic substitution to GSH, which protects cells by removing reactive oxygen species and regenerating *S*-thiolated proteins [30]. Intracellular total GSH levels in AML cells incubated with vitamin C peaked around 3 h, then declined, while the increase in incorporated [^35^S]-L-Cys peaked at 3 h and remained high. These results showed that [^35^S]-L-Cys transported into cells through cysteine uptake was incorporated and transferred intracellularly, strongly suggesting that the sulfhydryl transfer system is affected by vitamin C [30].

We therefore hypothesize that the biochemical pathway leading to thiol/disulfide redox regulation could be activated by vitamin C. Interestingly, of the proteins whose expressions changed by vitamin C treatment, immunoglobulin heavy chain binding protein (BiP, identical to Hsp70 chaperone) [19], like PDI, is also a multi-domain chaperone. BiP associated with the α-subunit of prolyl 4-hydroxylase (P4-H) by a disulfide bond [31]. P4-H consists of two distinct polypeptides, the catalytically more important α-subunit and the β-subunit, which is identical to the multifunctional enzyme, PDI [31]. Thus, BiP associated with the α-subunit of P4-H, which is another partner of PDI. The interaction of PDI with its substrates was due to a change in disulfide bonds, indicating that intrachain disulfide bonds between domains and substrates had been reduced [19]. Taken together, these results suggested that vitamin C oxidizes intracellular-reduced glutathione and affects disulfide bond formation in proteins [30].

Tropomyosin was also identified as a marker of the oxidative effect of vitamin C in NB4 cells. The spot corresponding to tropomyosin was positioned at an isoelectric point (pI) of approximately 5.0 and was attenuated in vitamin C-treated cells. In addition, a new spot having almost the same molecular weight was detected, which was positioned at a pI of 4.9 [19]. This new spot was also identified as tropomyosin, suggesting that post-translational chemical modification had affected its pI value. This result is consistent with previous data showing that the extracellular signal-regulated kinase (ERK)-mediated phosphorylation of tropomyosin-1 promoted cytoskeleton remodeling in response to oxidative stress [32]. The acidic shift of the spot with pI 5.0 to the phosphorylated tropomyosin spot by treatment with vitamin C was found to be abrogated by co-treatment with PD98059 [19], demonstrating that phosphorylation of tropomyosin was responsible for the observed acidic shift.

The significance of this observation may be related to differences in the regulation of the actomyosin contractile system in non-muscle cells as compared with that present in muscle cells. In addition, proteins that specifically reacted with sera from chronic myeloid leukemia patients included structural proteins, such as β-tubulin and tropomyosin isoforms [33]. Although the function of these proteins in myeloid leukemia needs further investigation, tropomyosin may have value as a leukemia-associated antigen and as a molecular target in antigen-based immunotherapy. In this regard, it is important to note that vitamin C causes a tropomyosin isoform to be modified during the immediate early response.

## 4. *In Vivo* Studies

Levine *et al.* reported that reaction products obtained from ascorbate *in vitro* are also found *in vivo* [34]. They showed that after i.v. injection, ascorbate baseline concentrations of 50–100 µM in blood and extracellular fluids peaked to >8 mM. After intraperitoneal injection, peaks approached 3 mM in both fluids. They hypothesized that *in vivo*, ascorbate was a prodrug for selective delivery of ascorbate radical and H_2_O_2_ to the extracellular space. Moreover, a regimen of daily pharmacologic ascorbate treatment significantly decreased growth rates of ovarian (*p* < 0.005), pancreatic (*p* < 0.05) and glioblastoma (*p* < 0.001) tumors established in mice [35]. In addition to inducing oxidative stress, high concentrations of ascorbic acid inhibited cell migration and the gap filling capacity of endothelial progenitor cells (EPCs) [36], and it has been reported that ascorbic acid inhibited corneal neovascularization in a rat model [37].

High concentrations of ascorbic acid also inhibited tumor growth in BALB/C mice implanted with sarcoma 180 cancer cells [38]. The survival rate increased by 20% in the group that received high doses of ascorbic acid, compared to controls. Gene expression studies from biopsy and wound healing analysis *in vivo* and *in vitro* suggested that the carcinostatic effect induced by high doses of ascorbic acid were related to inhibition of angiogenesis [39]. In addition, intraperitoneal administration of high doses of ascorbic acid quantitatively upregulated Raf kinase inhibitory protein (RKIP) and annexin A5 expression in a group of BALB/C mice implanted with S-180 sarcoma cancer cells. The increase in RKIP protein levels suggested that these proteins are involved in the ascorbic acid-mediated suppression of tumor formation [39]. Moreover, high doses of ascorbic acid (~1 mM) enhanced the apoptosis of cancer cells during co-treatment with paclitaxel, and the combinational treatment of paclitaxel and ascorbic acid ameliorated the side effects caused by paclitaxel in BALB/c mice implanted with or without sarcoma 180 cancer cells [40].

## 5. Clinical Studies

Cases of apparent responses of malignancies to intravenous vitamin C therapy have been reported, although they were reported without sufficient detail [15,41,42,43,44,45,46]. A recent study showed that oral administration of the maximum tolerated dose of vitamin C (18 g/day) produced peak plasma concentrations of only 0.22 mM, whereas intravenous administration of the same dose produced plasma concentrations approximately 25-fold higher. Larger doses (50–100 g) given intravenously resulted in plasma concentrations of approximately 14 mM [41].

Some case reports stated that high dose i.v. vitamin C has been used by Complementary and Alternate Medicine (CAM) practitioners [47]. Phase I evaluation of intravenous vitamin C in combination with gemcitabine and erlotinib in patients with metastatic pancreatic cancer data did not reveal increased toxicity with the addition of ascorbic acid [48]. No side effects were reported for most patients, while 59 were reported to have lethargy or fatigue out of 11,233 patients that received intravenous vitamin C in 2006 and 8876 in 2008 [47]. Overall, it was reported that high dose intravenous vitamin C did not appear to cause serious side effects in patients.

Another clinical study reported the depletion of L-ascorbic acid alternating with its supplementation in the treatment of patients with acute myeloid leukemia or myelodysplastic syndromes [49]. During the supplementation phase, patients received daily i.v. administration of vitamin C. A pre-therapy *in vitro* assay was performed for vitamin C sensitivity of leukemic cells from individual patients. Of the nine patients with the *in vitro* assay indicating their leukemic cells were sensitive to vitamin C, seven exhibited a clinical response, compared with none of the six patients who were insensitive to vitamin C.

## 6. Conclusions

Previous studies have shown that vitamin C is involved in the mechanism of action of the intracellular microenvironment (oxidation) state changes that improve therapeutic potential, including apoptosis and necrosis. Although it is difficult to postulate precise vitamin C-specific mechanisms at this time, identification of genes or proteins that are specifically regulated by vitamin C in certain cellular phenotypes could improve the efficacy of therapies.
